# RETRACTED: Nobel et al. Modern Subtype Classification and Outlier Detection Using the Attention Embedder to Transform Ovarian Cancer Diagnosis. *Tomography* 2024, *10*, 105–132

**DOI:** 10.3390/tomography10040040

**Published:** 2024-04-03

**Authors:** S. M. Nuruzzaman Nobel, S M Masfequier Rahman Swapno, Md. Ashraful Hossain, Mejdl Safran, Sultan Alfarhood, Md. Mohsin Kabir, M. F. Mridha

**Affiliations:** 1 Department of Computer Science and Engineering, Bangladesh University of Business and Technology, Dhaka 1216, Bangladesh; smnuruzzaman712@gmail.com (S.M.N.N.); masfequier.cse.bubt@gmail.com (S.M.M.R.S.); ashrafulhossainwork@gmail.com (M.A.H.); 2 Department of Computer Science, College of Computer and Information Sciences, King Saud University, P.O. Box 51178, Riyadh 11543, Saudi Arabia; sultanf@ksu.edu.sa; 3 Superior Polytechnic School, University of Girona, 17071 Girona, Spain; u1985702@campus.udg.edu; 4 Department of Computer Science, American International University-Bangladesh, Dhaka 1229, Bangladesh; firoz.mridha@aiub.edu

The *Tomography* Editorial Office retracts the article “Modern Subtype Classification and Outlier Detection Using the Attention Embedder to Transform Ovarian Cancer Diagnosis” [1], cited above.

Following publication, concerns were brought to the attention of the publisher regarding unauthorized use of propriety data presented within this article [1].

Adhering to our complaints procedure, an investigation was conducted that confirmed that appropriate permission to publish these data was not obtained and that this publication breached the embargo period as set out by the Kaggle competition “UBC Ovarian Cancer Subtype Classification and Outlier Detection (UBC-OCEAN)”, hosted by the University of British Columbia (UBC), Canada. As retroactive permission could not be obtained, the Editorial Office and Editorial Board have decided to retract this article as per MDPI’s retraction policy (https://www.mdpi.com/ethics#_bookmark30) and in line with the Committee on Publication Ethics retraction guidelines (https://publicationethics.org/retraction-guidelines).

This retraction was approved by the Editor-in-Chief of the journal *Tomography*. 

The authors did not provide a comment on this decision. 
